# Increased copeptin levels in metabolic syndrome from a Romanian population

**Published:** 2016

**Authors:** M Vintilă, ML Gheorghiu, A Caragheorgheopol, N Baculescu, C Lichiardopol, C Badiu, M Coculescu, F Grigorescu, C Poiană

**Affiliations:** * ”Carol Davila” University of Medicine and Pharmacy, Bucharest, Romania; ** ”C.I. Parhon” National Institute of Endocrinology, Bucharest, Romania; *** University of Medicine and Pharmacy Craiova, Romania; **** Laboratory of Molecular Endocrinology, IURC, UMR204, NUTRIPASS (IRD, University of Montpellier, SuprAgro); † Passed away in March 2016

**Keywords:** copeptin, arginine vasopressin, metabolic syndrome, insulin resistance

## Abstract

**Rationale:** Arginine vasopressin (AVP) is secreted under conditions of water deprivation. Since AVP has a low half-life in the plasma, the C-terminal fragment of AVP-precursor (copeptin) was used to estimate the AVP levels. High copeptin levels increase the risk for the development of diabetes mellitus.

**Aim:** This study was aimed to measure copeptin levels in the metabolic syndrome (MetS) in Romanians using a competitive enzyme immunoassay.

**Methods and results: **Patients prone to present MetS (n = 63) were compared to controls (n = 42). In the MetS group, the syndrome was confirmed in 93.6%. Affected patients displayed 85.7% obesity and insulin resistance (HOMAIR of 4.9 ± 0.4 versus 1.1 ± 0.8 in controls). Low HDL-cholesterol was less represented (47.5%). Copeptin levels were 0.6 ± 0.0 in MetS versus 0.42 ± 0.0 ng/ mL in controls (P < 0.004). Higher copeptin (0.79 to 1.83 ng/ mL) was associated with MetS, P < 0.0018, OR 20, 95%CI [3.03 – 131.7]. In ANOVA, high copeptin was equally explained by MetS or obesity (P < 0.05,α = 3.8). The best correlation was found with high triglyceride levels (P < 0.013,α = 6.3) while the correlation with HOMAIR remained not significant.

**Discussion:** These data indicated a concordant correlation between increased copeptin and MetS or its components. In the light of epidemiological data, indicating that more than 50% of the European population has a lower daily water intake and a fraction of 25% displaying high copeptin, our data further sustained that copeptin may be a good biomarker for MetS and/ or obesity, which should be further investigated with other members of the osmoregulation pathway at both pathogenesis and genetic levels.

## Introduction

Metabolic syndrome (MetS) is an important public health indicator, in relation with cardiovascular mortality and all cause mortality of populations [**1**,**2**], affecting around one-fourth of the world population [**3**]. It clusters a series of cardiometabolic risk factors, involving abdominal obesity, hypertension, dyslipidemia, and hyperglycemia [**4**]. Insulin resistance is an essential characteristic of the syndrome, however the etiology of MetS is still imprecisely known, and different pathophysiological perturbations are described, some reflected by diverse biomarkers, such as C-reactive protein, leptin or copeptin [**5**,**6**].

Arginine vasopressin (AVP), a neurohormone released from the neurohypophysis, maintains fluid homeostasis by inducing water reabsorption in the kidney. AVP also affects osmoregulation and produces vasoconstriction [**7**]. Besides these well known functions, AVP plays roles in ACTH secretion, lipid metabolism, and glucose homeostasis, influencing hepatic gluconeogenesis and glycogenolysis, and insulin and glucagon release from pancreatic cells as the function of extracellular glucose level [**8**-**10**].

The measurement of the C-terminal fragment of AVP precursor (copeptin) was used to estimate the AVP secretion. Copeptin is secreted in equimolar amounts with AVP. Therefore, it reflects its release and can serve as a reliable surrogate marker for circulating levels of AVP [**11**,**12**]. Copeptin measurement became a useful tool for clinical practice, since AVP is a short-lived peptide, unstable in isolated plasma and its measurement in biological fluids is difficult to manage [**11**]. 

More recently, copeptin was recognized as a good biomarker of MetS[**6**,**13**]. In several studies, Copeptin was shown to be associated with insulin resistance, obesity, and metabolic disturbances [**14**-**16**]. Higher levels of copeptin were associated with an increased risk of development of diabetes mellitus independently of other recognized risk factors [**17**,**18**]. These data support the hypothesis that AVP plays a role in metabolic disorders.

The aim of this study was to investigate the association of serum copeptin with MetS and define potential correlations with its components in the Romanian population. 

## Subjects, materials, and methods

**Subjects**. Patients were recruited in the frame of the MEDIGENE European project (FP7-279171) intended to investigate genetic and environmental factors of insulin resistance syndrome in the Mediterranean populations (www.medigene-fp7.eu). All subjects were Caucasians, born in Romania and having the birthplace in the same geographical region for index cases and 2 generations upstream family members. Patients and controls were recruited in accordance with the Helsinki Declaration (as revised in 1983),an informed consent being obtained from all the patients. Protocol was approved by the Ethics Committee of “Carol Davila”University of Medicine and Pharmacy, Bucharest (Romania). All the subjects were declared to MESR in France as MEDIGENE-1 collection (CODECOH DC-2014-2226) with an approval for the import export data and biological material. 

Participants were included in the study either as controls from the general population or as patients suspected to present MetS. Criteria for the recruitment were often obesity and dyslipidemia. Diagnosis of MetS was defined according to the National Cholesterol Education Program (NCEP) Adult Treatment Panel-III (ATP-III) criteria[**19**]. Anthropometric parameters (height, weight, waist, and hip circumferences) were measured in each subject. Complicated medications were excluded, and subjects were maintained on a patient-choice-diet (300 g carbohydrate daily). Blood pressure was determined by using a sphygmomanometer after 10 minutes of rest in supine position. Glucose intolerance (IGT) and impaired fasting glucose (IFG) were defined by the 2006 American Diabetes Association criteria[**20**]. Control subjects (over 30 years old) were recruited with the occasion of the annual check up in the same institution, and apparently in good health and submitted to the same protocol of investigation. 

**Biochemical assays**. Serum samples were obtained after an overnight fast. The analyses of glucose, total cholesterol, low-density lipoprotein (LDL) cholesterol, high-density lipoprotein (HDL) cholesterol, triglycerides, insulin, C-reactive protein were measured by using standardized kits. Homeostasis model assessment of insulin resistance (HOMAIR) was calculated by fasting glycemia (mmol/L) x insulin (µUI/mL)/22.5. The cut-off value for insulin resistance (IR) patients was of 1.7 (mean + 1 SEM) of controls. 

For copeptin, serum samples were collected and stored at -80ºC until measurement. Copeptin was measured by using a commercially competitive enzyme immunoassay (Phoenix Pharmaceuticals, Inc). The minimum detection limit was 0.1 ng/mL. All samples were assayed in duplicate. Intra- and inter-assay variations were both less than 20%.

**Data and statistical analysis**. Statistics were performed by using StatView 5.1 and SAS (Abacus Concepts, Berkeley, CA) as described [**21**]. Numerical variables were expressed as mean ± SEM and nominal variable data as percentages. Two groups (control and MetS) were analyzed with Mann-Whitney statistical test while nominal variables were compared by chi-squared test. Preliminary correlations were searched by Spearman correlation test. Multivariate analysis was performed in ANOVA and logistic regression. The significance level was set at 0.05.

## Results

In this study, we included 105 subjects, among whom women (79%) represented a greater proportion. Following the ATP-III definition, in the group recruited for the MetS, the syndrome was confirmed in 93.6% (n = 59). Three patients presented only 1 or 2 criteria. None of subjects of the control group received the diagnosis of MetS. However, 23.8% of the controls presented 1 criterion and another 16.6% 2 criteria. If the prevalence of MetSin the totality of these clinical series was estimated, MetS was present in 56.2% of the whole population with no significant difference between man and women (52.1% and 56.2%, P < 0.36, respectively). Clinical and laboratory features of this population are indicated in **Table 1**.

**Table 1:** Clinical and laboratory features of MetS and control population in Romanians. Data are presented as mean ± SEM and controls and MetS groups were compared by using Mann-Whitney test for numerical variable and chi2 for nominal variable

	CTR	MetS	P value*
*n*	42	63	NA
Gender (F/ M)	31/ 11	51/ 12	NA
Age (years)	47.9 ± 1.7	50.9 ± 1.4	NS
BMI (kg/ m2)	22.8 ± 0.4	35.7 ± 0.8	<0.0001
Waist (cm)	81.8 ± 1.2	110.7 ± 1.8	<0.0001
Obesity (%)†	2.3	85.7	<0.0001
SBP (mmHg)	117.1 ± 2.0	145.0 ± 2.6	<0.0001
DBP (mmHg)	73.2 ± 1.4	87.6 ± 2.1	<0.0001
Hypertension (%)‡	26.1	93.6	<0.0001
Fasting Glucose (mmol/ L)	4.7 ± 0.0	6.8 ± 0.2	<0.0001
Fasting insulin (µU/mL)	5.4 ± 0.4	16.5 ± 1.4	<0.0001
Hyperglycemia (%)c	0.0	79.3	<0.0001
HOMA IR	1.1 ± 0.8	4.9 ± 0.4	<0.0001
Insulin resistance (%)§	9.5	88.8	<0.0001
Triglycerides (mmol/ L)	0.9 ± 0.0	1.9 ± 0.1	<0.0001
HDL-cholesterol (mmol/ L)	0.7 ± 0.0	0.5 ± 0.0	<0.0001
High Triglycerides (%)c	19.0	85.7	<0.0001
Low HDL (%)c	9.5	44.4	<0.0001
CRP (mg/ dL)	0.2 ± 0.0	1.6 ± 0.5	NS
Copeptin (ng/ mL)	0.42 ± 0.0	0.6 ± 0.0	0.004
Smokers (%)	19.5	22.0	NS
*NA stands for non applicable, NS stands for non significant; †Obesity was considered by BMI and waist circumference calculated separately between men and women; ‡hyperglycemia, high triglycerides and low HDL were considered taking into account treatment or pre-diagnosed type 2 diabetes; §insulin resistance was considered as function of HOMAIR values with a cut-off of 1.7.			

Patients with MetS were older than the controls, but not significantly. They most frequently displayed obesity (85.7%) with a 1.6 fold increase in the BMI. Central obesity, hyperglycemia, high triglycerides, low HDL-cholesterol, and hypertension were present in 88.1, 83.0, 88.1, 47.5, 94.9%, respectively. Three patients in the MetS group were not confirmed (< 3 criteria); therefore, they were excluded from further statistics. Patients with MetS were more insulin resistant (88.8%) with HOMAIR values of 4.9 ± 0.4. CRP levels were not different between MetS and controls. These data indicated a clear diagnosis of MetS in the Romanian population and a good selection of patients entering the protocol. 

**Fig. 1:**
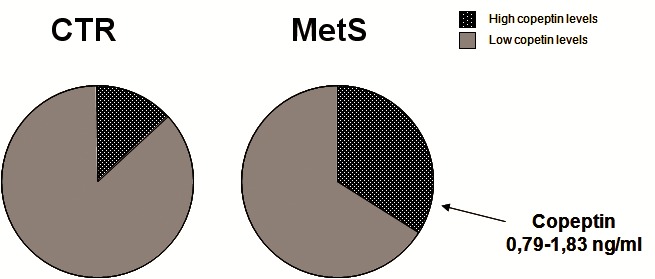
Distribution of copeptin levels in control and MetS groupsThe highest values of copeptin (considered by centiles) were present in 9.5% of controls and in 33.9% of patients with MetS

**Copeptin**. The mean copeptin levels in patients with MetS were 0.6 ng/mL versus 0.42 ng/mL in controls (P < 0.004, Mann-Whitney). The association remained significant when adjusted for age and sex (P < 0.03). There was no difference between males and females for the copeptin level (0.39 ± 0.37 ng/mL, versus 0.57 ± 0.41ng/mL, P<0.06) for the whole group. There was a wide variability of copeptin levels from 0.06 to 1.83 ng/mL. To better understand the significance of these values in MetS we considered the centiles of copeptin. The highest values of copeptin (0.79 to 1.83 ng/mL) were present in 9.5% of the controls and in 33.9% of the patients with MetS (**Fig. 1**). In the logistic regression, this category of copeptin values was associated with MetS, with P < 0.0018, OR 20, 95%CI [3.03 – 131.7]. There were no major variations in the OR when the association was adjusted for various components of MetS.

To understand the potential correlation between copeptin levels and various parameters of MetS, we performed the Spearman correlation test. Significant values were found regarding age (P < 0.0012, Rho 0.32), BMI (P < 0.0095, Rho 0.26) and HDL-cholesterol values (P < 0.0062, Rho – 0.2). 

In ANOVA, copeptin levels were well explained by the presence of MetS (P < 0.029, λ = 4.8) from values of 0.42 ± 0.06 to 0.60 ± 0.05 ng/mL. Obesity (central obesity) was also a good explanation, with P < 0.05,α= 3.8. Among other parameters, triglyceride levels were well correlated in ANOVA with P < 0.013,α= 6.3. For insulin or HOMAIR values, results were at the limit of statistical significance. Thus, insulin resistance explained the copeptin levels with P < 0.051,α= 3.8. These data indicated a correlation between the increased levels of copeptin and MetS and its components in a concordant manner, patients with increased levels being more obese, with higher triglyceride levels and more insulin resistant. 

## Discussion

In this study, we presented evidence for an increased level of copeptin in MetS in a Romanian population, as a surrogate measure of AVP levels. Increased levels of copeptin were also correlated with obesity, dyslipidemia, and insulin resistance. These data confirmed a previous observation in other European populations and sustained the interesting hypothesis that hyper-secretion of AVP would be related with an increased risk of type 2 diabetes or MetS in the general population [**22**]. Thus, copeptin may be used as a biomarker in MetS and cardiovascular complications in clinical trials or in more extended epidemiological studies. 

The prevalence of the MetS in the entire clinical series of 105 subjects was of 56.2%. Obviously, this did not reflect the prevalence of MetS in the general population. In population-based studies, MetS was found in < 30%. However, the prevalence of 93.6% in the group suspected for MetS indicated a good recruitment based on our criteria of inclusion. Thus, only in 3 patients, the presence of ≥ 3 ATP-III criteria was not confirmed. 

In our population of MetS, the most frequent component was hypertension (94.9%) and the less frequent was decreased levels of HDL-cholesterol (47.5%). Since HDL-cholesterol was strongly related with insulin resistance, these data might explain why the correlation with copeptin did not reach a statistical significance, although it appeared as a trend. In contrast, a better correlation was found between the increased level of copeptin and obesity. This feature was further confirmed in linear regression between copeptin levels and BMI with P < 0.034, R2 = 0.045. 

This study presented quite a variation of copeptin levels. However, the mean values of 0.4 and 0.6 ng/ml fit in the range previously reported in other populations. The copeptin level was reported as being higher in men [**23**]. In our study group, we did not find a difference between men and women. Obviously, this was explained by the higher prevalence of women in this clinical series and very likely, our data reflected better the women population. No correlation was found with age, concordant with data from the literature [**24**], although the initial investigation with the Spearman test found a correlation with age.

Certainly, our study had several limitations, particularly related to the still small size of our population and the high percent of women. The variation of copeptin levels might be explained by still high variability in the biochemical measurement and the potential variation in plasma osmolarity. However, one can assume a plasma osmolarity in normal range, as the participants came from outpatient care, had normal natremia, and did not present clinical reasons or signs for dehydration or overhydration.

Data presented in this study should be analyzed from the perspective of defining more biomarkers for MetS in human populations. Copeptin reflected AVP release [**12**] and was used more and more as a surrogate marker for AVP in various medical conditions. In recent years, an increasing number of studies have evaluated the role of AVP and the activation of hypothalamic-pituitary adrenal axis by AVP in insulin resistance and metabolic abnormalities. The association between copeptin and obesity has been previously described [**6**,**13**]. Enhörninget al.reported that copeptin independently predicted abdominal obesity and diabetes mellitus [**25**]. Higher copeptin levels were also reported in obese women with polycystic ovary syndrome (PCOS), as compared to non-obese PCOS and control women [**26**]. It is well known that PCOS is associated with insulin resistance and PCOS-women who carry a 2-4 fold higher risk of developing MetS than non-PCOS women [**27**].

The mechanism through which copeptin levels correlate with obesity is not completely understood. The effect of AVP on ACTH release through pituitary V1b receptors with consecutive increase in glucocorticoid levels is speculated [**6**]. Moreover, it seems that ACTH release stimulated by AVP is resistant to cortisol feedback, as opposed to CRH mediated release [**28**]. It should be mentioned that the implication of the AVP pathway in the pathogenesis of MetS and insulin resistance is complex, perhaps involving not only the AVP secretion but also genetic and non-genetic effects of both V1a and V1b receptors in the liver and pancreas. Several studies showed that AVP stimulates the production of triglycerides in hepatocytes [**29**] and enhanced lipid metabolism with low triglyceride levels was found in V1a receptor knock-out mice [**30**]. Along this line, our laboratory is currently investigating the genetic variation of AVPR1A in MetS in the frame of European programMEDIGENE, for which the use of copeptin measurements as biomarker may be of crucial importance. 

In conclusion, this study presented evidence for an increased level of copeptin in MetS in a Romanian population and good and concordant correlation with components of this syndrome, including obesity. 

**Acknowledgments**

The authors are grateful to the clinicians who contributed to the recruitment of patients.

This paper is dedicated to Professor MihailCoculescu, an inspiring teacher and mentor, who passed away before this paper was published.

**Sources of funding**

This study received financial support from EC as European project FP-7 MEDIGENE (279171) and through the project entitled “CERO – Career profile: Romanian Researcher”, grant number POSDRU/159/1.5/S/135760, cofinanced by the European Social Fund for Sectoral Operational Programme Human Resources Development 2007-2013.

**Disclosures**

None.
